# Research on the height of primary school students in Zhuzhou and analysis of influencing factors of short stature

**DOI:** 10.3389/fped.2025.1448309

**Published:** 2025-02-18

**Authors:** Xiao-min Ye, Qiong Tang, Yi-can Yang, Xiang-Lan Wen

**Affiliations:** Department of Children Health Care Center, Zhuzhou Hospital Affiliated to Xiangya School of Medicine, Central South University, Zhuzhou, Hunan, China

**Keywords:** growth curve, height, incidence of short stature, influencing factors, school-aged children

## Abstract

**Objective:**

To construct a growth curve for children aged 6–12 years residing in Zhuzhou, assess the height distribution among local elementary school students, and analyze the factors contributing to short stature.

**Methods:**

We measured the heights of children from 110 elementary schools in Zhuzhou using cluster sampling. A total of 106,864 samples of children aged 6–12 years were collected and divided into 25 age groups, each spanning three months. The Lambda-Mu-Sigma (LMS) method was employed to calculate the 3rd, 10th, 25th, 50th, 75th, 90th, and 97th percentiles of height for each age group. Children below the 3rd percentile according to the national growth curve made in 2005 were considered to be short stature. The heights of boys and girls in Zhuzhou were compared with the national average heights from 2005. The height growth curve was constructed using the curve estimation function of SPSS software and the influencing factors for the prevalence of short stature were identified using logistic regression analysis based on data obtained from parental questionnaires.

**Results:**

The prevalence of short stature among children aged 6–12 years in Zhuzhou was found to be 3.97%. Boys exhibited a significantly higher incidence of short stature at 4.53% compared to girls at 3.37%. Children residing in suburban areas showed a notably higher prevalence of short stature (5.80%) compared to their urban counterparts (3.67%), with these disparities proving statistically significant. Logistic regression analysis identified several contributing factors for short stature in this population, including low birth weight, inadequate daily physical activity (less than 0.5 h), father's educational qualification of junior college level or below, short stature of parents, insufficient sleep duration, and male gender.

**Conclusion:**

To improve children's height in Zhuzhou, it's important for them to exercise for over 30 min daily and sleep for more than 10 h each night. Regular monitoring of growth and nutrition is crucial. Health education for families with less educated fathers and early intervention for children with short-statured parents are also key strategies.

## 1 Background

Height serves as a primary indicator of physical growth in children, playing a crucial role in assessing their nutritional status and overall health. Adherence to established height standards enables the identification of growth retardation in children, which remains a significant public health concern globally and within specific regions. Regional studies within our country have demonstrated varying rates of growth retardation, underscoring disparities in height distribution (1). Notably, there are currently no research reports on the height status incidence rates or influencing factors of short stature among school-aged children in Zhuzhou.

This study utilizes SPSS software to conduct curve fitting analysis on height data collected from children aged 6–12 years in Zhuzhou. The aim is to characterize the regional height distribution in this age group and compare it with national data. Additionally, through parent surveys, the study focuses on factors that contribute to short stature among children in the area. These findings aim to provide a theoretical foundation for developing targeted health interventions aimed at reducing the incidence of short stature in this city.

## 2 Materials and methods

### 2.1 Source of materials

The data analyzed for this study were obtained from the 2022 Zhuzhou City general survey of physical growth among school-aged children aged 6–12. The survey encompassed 115,227 children from 110 elementary schools, employing a cluster sampling approach. Data collected included information on gender, age, and height. Exclusion criteria were applied to children younger than 6 or older than 12 years and 2 months; non-residents of Zhuzhou; and those with specific conditions such as limb disabilities, cardiopulmonary diseases, chromosomal disorders, congenital diseases, skeletal development disorders, or severe intellectual disabilities. Ultimately, the study included 106,864 children comprising 55,734 boys and 51,130 girls. Among them, 91,633 attended urban schools within Zhuzhou (47,714 boys and 43,920 girls), while 15,230 attended suburban schools (8,020 boys and 7,210 girls). Children were categorized into 25 age groups based on gender, with each group representing a three-month age increment from 6 years up to 12 years.

### 2.2 Measurement of height

Height measurements were conducted uniformly by trained staff using standardized instruments that were calibrated according to established protocols. The specific measurement methods adhered to guidelines outlined in Pediatric Health Care (4th edition) (2).

### 2.3 Constructing the height percentile curve using SPSS software

Human height data are skewed distribution data, thus initially requiring the application of the coefficient of skewness-median-coefficient of variation (CSMCV, LMS) (3, 4). Data normalization was achieved through Box-Cox transformation, typically performed using LMS software, or mathematical formulas. This transformation estimates the parameter λ (often using maximum likelihood estimation), yielding an optional *λ* value (denoted as L). Age, serving as the independent variable for grouping in anthropometric data, was utilized to fit L values across all age groups into a smooth curve. Subsequently, the M (median) and S (coefficient of variation) values for each age group were derived and integrated into the M curve and S curve (5).

Using the LMS formula: C_100a_(t) = M(t)[1 + L(t)S(t)Z*α*]^[1/L(t)]^, the percentile values corresponding to specific Z*α* values (where Z*α* represents normal deviations for tail areas P3, P10, P25, P50, P75, P90, and P95 with Z values −1.88, −1.28, −0.67, 0, 0.67, 1.28, and 1.88 respectively), were calculated (6). Here, C100a represents the percentile corresponding to Z*α*. The variables t denotes age, M(t), L(t), and S(t) represent the corresponding M, L, and S values, respectively. By substituting these values into formula (1), the respective percentiles can be calculated. This calculation methodology follows the approach details by Lei (7).

In this study, the L, M, and S curves were not graphically plotted; the L, M, and S values for each age group were computed using described formula, thereby determining the corresponding percentile for each age category. This study's curve fitting process utilized SPSS 23.0 software. Initially, organized age and percentile height data were grouped by age and imported into the SPSS data window. The ‘Analysis' dropdown menu was assessed where ‘Regression’ and ‘Curve Estimation’ options were selected to determine the most appropriate fitting model. Subsequently, percentile values for the height of boys across each age group —specifically the 3rd (P3), 10th (P10), 25th (P25), 50th (P50), 75th (P75), 90th (P90), and 97th (P97) percentiles — were imported and used to generate the respective curves based on the selected model. Final adjustments to the curves were made using the ‘Apply Template’ function following the method described by Chang (8).

### 2.4 Evaluation of curve fitting effect

Four evaluation methods were utilized in order to assess the effectiveness of the constructed curves. Firstly, the curve fitting models were evaluated based on the coefficient of multiple correlation (R) where a value closer to 1 indicates a better fit (9). Secondly, the relative error between measured values and fitted curve values was computed; a smaller relative error indicates a more accurate fit. Thirdly, the mean absolute percentage error (MAPE) was calculated using the formula MAPE = (1/n) * *Σ* (|measured value—fitted value|/measured value) * 100, where *n* is the sample size, with a lower MAPE value indicating better accuracy fit (10). Finally, the intra-class correlation coefficient (ICC) was determined to assess consistency between measured values and fitted values, with an ICC >0.9 indicating high consistency and a significance level (*P* < 0.05) indicating statistically significant differences (11).

### 2.5 Calculation of the incidence of short stature and logistic regression analysis of influencing factors

The diagnostic criterion for short stature in this study is defined as a height below the 3rd percentile (P3) of the 2005 national height growth curve for nine cities. To identify the potential influencing factors of short stature, parental questionnaires were distributed concurrent with the census and voluntarily completed, resulting in 26,757 valid responses. The questionnaire included variables such as gender, gestational age (preterm, full-term, and post-term), birth weight (< 2.5 kg, 2.5–4 kg, and >4 kg), history of allergies, daily duration of physical activity (< 0.5 h, 0.5–1 h, and >1 h), sleep duration at night (< 8 h, 8–10 h, >10 h), mother's height, father's height, father's educational background (junior college and below, bachelor's degree, and graduate degree), and mother's educational background (junior college and below, bachelor's degree, and graduate degree). Binary logistic regression analysis was utilized to identify factors influencing the incidence of short stature. Among them, 15,010 parent questionnaires provided valid responses regarding annual household income, which was categorized into four groups based on the 2022 Zhuzhou City Statistics Bureau data: below 48,000 yuan, 48,000–120,000 yuan, 120,000–240,000 yuan, and above 240,000 yuan.

### 2.6 Statistical analysis

The short stature rates between urban and suburban areas and between boys and girls were compared using the Chi-square test. Binary logistic regression, stature in combination with the parental questionnaire, was used to analyze the influencing factors of the prevalence of short stature.

## 3 Results

### 3.1 Height curve fitting results

**Figure 1:**
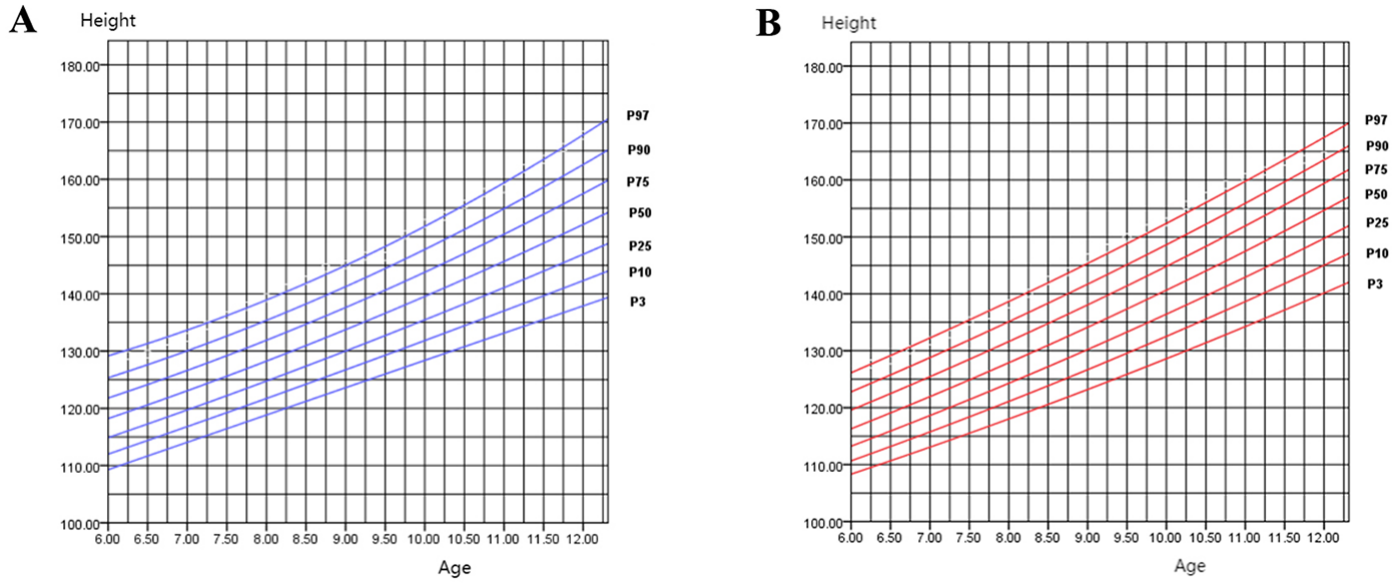
**(A)** Height percentile curve for boys aged 6–12 in Zhuzhou city; **(B)** height percentile curve for girls aged 6–12 in Zhuzhou city.

Figures 1A,B depict the growth curves for boys’ and girls’ height in Zhuzhou, constructed using SPSS software. Each curve in the diagrams includes seven lines arranged vertically, representing the percentiles P3, P10, P25, P50, P75, P90, and P97 respectively. These curves exhibit smooth trajectories and consistent trends, reflecting highly satisfactory fitting results.

Table 1 presents the parameters of the fitting models for boys’ percentile curves, where R2 values exceed 0.9, indicating significant statistical reliability of the constructed models (*P* < 0.001). Among the models evaluated, the quadratic and cubic models exhibit the highest R values. The linear model also shows a notable R value, possibly due to capturing the gradual linear growth phase typical in children aged 6–12. Based on the methodology outlined by Chang et al., a quadratic model was selected to construct the boys’ height percentile curve, while a regression model was chosen for the girls’ height percentile curve (8).

**Table 1:** Parameters for children's growth curve fitting model.

Male				Female				*P*-value
Model	R2	AdjustedR2	F	Model	R2	AdjustedR2	F	
Linear	0.996	0.996	6,009.831	Linear	0.750	0.750	149,728.885	<0.001
Quadratic	0.996	0.996	2,875.588	Quadratic	0.751	0.751	75,337.945	<0.001
Cubic	0.996	0.996	2,877.081	Cubic	0.751	0.751	75,337.945	<0.001
Logistic	0.995	0.995	4,741.408	Logistic	0.755	0.755	153,234.830	<0.001

R, correction coefficient; R2, determination coefficient; SE, standard error.

### 3.2 Height curve fit effect test

Using boys as an example to assess the fitting quality of the height curve, Table 2 provides the fitted values and measured values of boys’ heights at P10, P50, and P90 percentiles across each age group, calculated using the LMS method. The relative error of the fitted values at these percentile for each age group is consistently below 1%.

**Table 2:** Measured values, fitted values, and relative error for heights P10, P50 and P90 of boys aged 6–12 in Zhuzhou city.

Age (year)	*n*	P10			P50			P90		
		Fitted value	Actual value	Relative error	Fitted value	Actual value	Relative error	Fitted value	Actual value	Relative error
6	63	112	112.5	−0.47	118.1	118.8	−0.65	124.8	125.2	−0.4
6.25	992	113.2	112.9	0.27	119.4	118.7	0.54	126.1	125.6	0.5
6.5	1,545	114.4	114.2	0.17	120.6	120.3	0.26	127.4	126.9	0.5
6.75	2,478	115.6	115.4	0.21	122.0	121.7	0.23	128.7	128.2	0.5
7	2,423	116.9	117	−0.08	123.3	123.4	−0.08	130	130	0
7.25	2,474	118.1	117.8	0.25	124.6	124.6	0	131.4	131.5	−0.1
7.5	2,524	119.3	119.3	0	125.9	126.0	−0.06	132.8	133.6	−0.8
7.75	2,456	120.6	120.4	0.14	127.3	127.3	0.01	134.2	134.2	0
8	2,462	121.8	121.8	0.01	128.7	128.7	−0.01	135.6	135.8	−0.2
8.25	2,528	123.1	123.4	−0.28	130.0	130.4	−0.31	137.1	137.5	−0.4
8.5	2,447	124.3	124.9	−0.43	131.4	131.3	0.1	138.6	139.6	−1
8.75	2,527	125.6	125.6	0.01	132.8	133.0	−0.09	140.2	140.3	−0.1
9	2,519	126.9	127.3	−0.35	134.3	134.1	0.14	141.7	141.4	0.3
9.25	2,827	128.2	128.3	−0.1	135.7	135.7	0.03	143.3	143.3	0
9.5	2,760	129.4	129.9	−0.34	137.2	137.6	−0.3	145	145.3	−0.3
9.75	2,554	130.7	129.2	1.2	138.7	139.4	−0.55	146.6	146.1	0.5
10	2,439	132	132	0.01	140.2	139.9	0.21	148.3	148.1	0.2
10.25	2,462	133.4	133.3	0.04	141.7	141.5	0.13	150	149.5	0.5
10.5	2,309	134.7	134.9	−0.2	143.2	143.1	0.07	151.8	151	0.8
10.75	2,223	136	135.4	0.41	144.8	144.2	0.39	153.5	153.3	0.2
11	2,195	137.3	137.6	−0.18	146.3	145.8	0.37	155.3	154.8	0.5
11.25	2,299	138.7	139.1	−0.35	147.9	148.0	−0.09	157.2	157.8	−0.6
11.5	2,291	140	140.3	−0.22	149.5	149.6	−0.07	159	159.5	−0.5
11.75	2,141	141.4	140.8	0.39	151.2	151.4	−0.14	160.9	160.8	0.1
12	1,796	142.7	142.9	−0.1	152.8	153.0	−0.12	162.9	163.3	−0.4

Table 3 displays the MAPE values comparing different percentile fitted values with measured values across age groups. The MAPE values for P3 to P97 are all below 1%, suggesting highly accurate curve fitting results.

**Table 3:** MAPE between percentile estimates and measured values.

	P3	P10	P25	P50	P75	P90	P97
MAPE	0.54	0.25	0.16	0.20	0.22	0.27	0.38

In Table 4, the Intra-class Correlation Coefficient (ICC) is presented for fitted values of boys’ heights at P10, P50, and P90 percentiles compared to actual values across ages 6–12. All ICC values are close to 1, with *P* < 0.001. These findings collectively affirm the effectiveness of the curve fitting process.

**Table 4:** Consistency comparison of measured and fitted values for heights P10, P50, and P90 Among boys in different Age groups.

	Intra-class correlation coefficient (single measurement)	95% confidence interval	F	Significance
P10	1.0	0.999–1.0	4,867.67	<0.001
P50	1.0	1.0–1.0	14,710.28	<0.001
P90	1.0	1.0–1.0	17,078.72	<0.001

### 3.3 Comparison of Zhuzhou height curve with national levels

**Figure 2:**
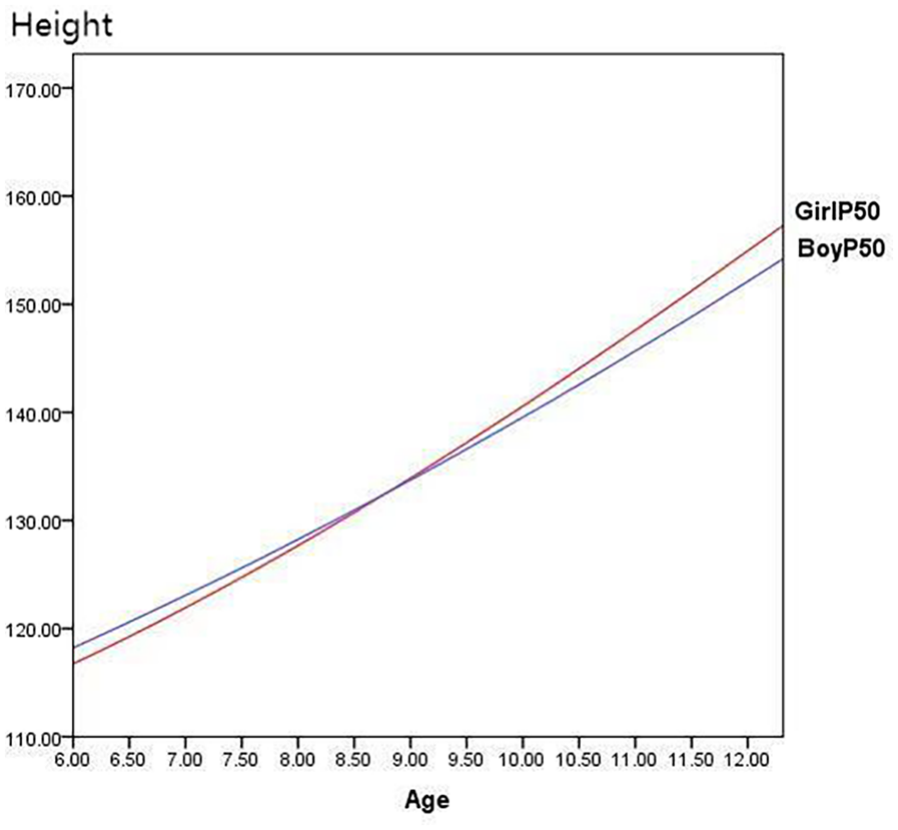
Comparison of P50 height between boys and girls in Zhuzhou city.

**Figure 3:**
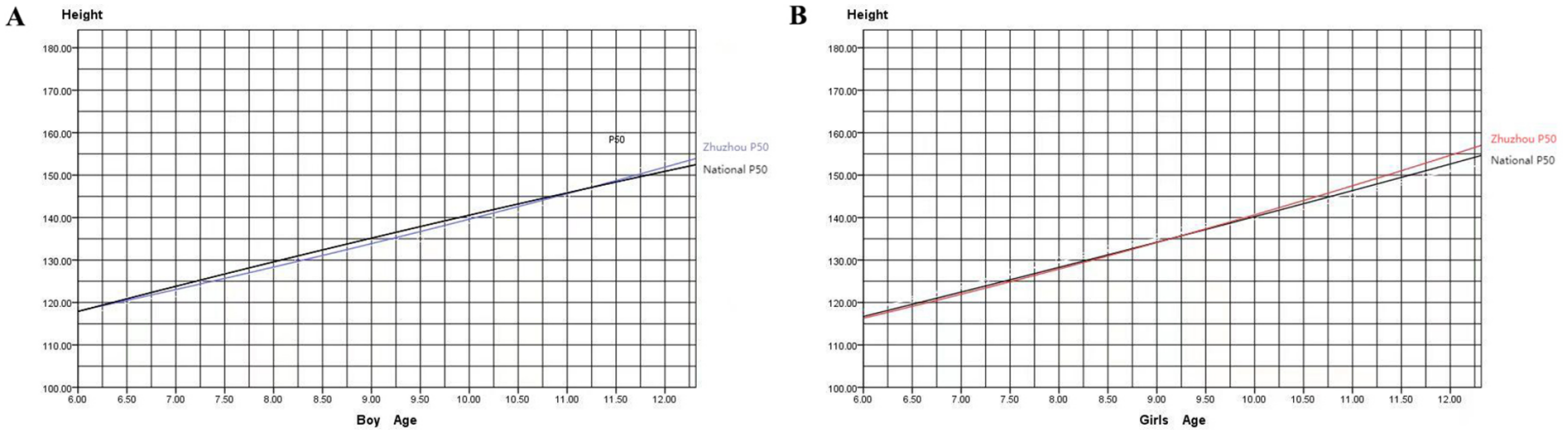
**(A)** Comparison of heights between boys aged 6–12 in Zhuzhou city and the national average; **(B)** comparison of heights between girls aged 6–12 in Zhuzhou city and the national average. Note: The blue curve represents Zhuzhou city boys, the red curve represents Zhuzhou city girls, and the black curve represents the 2005 national growth curve.

Figure 2 illustrates that girls in Zhuzhou surpass the boys in height at approximately 8.75 years of age at P50 percentile level. Figures 3A,B compare the heights of boys and girls in Zhuzhou with the national average heights from 2005. It is observed that boys in Zhuzhou are slightly below the national average from ages 6–11 but exceed it after age 11. Girls in Zhuzhou are slightly below the national average from ages 6–9.5 but exceed it after 9.5 years of age.

### 3.4 Analysis of the incidence of short stature and factors influencing short stature

In Zhuzhou, the overall incidence of short stature among children aged 6–12 is 3.98% (4,248/106,864). Specifically, the urban incidence is 3.67% (3,365/91,633), while the suburban incidence is higher at 5.80% (883/15,230). Table 5 highlights that in both urban and suburban areas, boys have significantly higher incidences of short stature compared to girls, with the suburban incidence also significantly higher than urban incidence, indicating statistically significant differences.

**Table 5:** Comparison of short stature rates Among children aged 6–12 in zhuzhou city.

	Male (short stature/total)	Female (short stature/total)	X^2^_1_	*P*-value_1_
Total	4.53% (2,526/55,734)	3.37% (1,722/51,130)	94.607	<0.001
Urban area	4.2% (2,005/47,714)	3.1% (1,360/43,920)	78.935	<0.001
Suburban area	6.5% (521/8,020)	5.0% (362/7,210)	15.119	<0.001
X^2^_2_	83.532	<0.001		
*P*-value_2_	70.465	<0.001		

Note: 1 comparing between boys and girls, 2 comparing between urban and suburban areas.

Binary logistic regression analysis detailed in Table 6 demonstrates that gestational age (full-term and post-term) were not included in the model equation. The Hosmer-Lemeshow test showed a chi-squared value of 5.472 with a *P*-value of 0.706 (> 0.05), indicating a good model fit. The analysis identifies independent risk factors for short stature including birth weight <2.5 kg, daily physical activity <0.5 h, father's education background of junior college or below, being male, and underweight (body mass index <P5). Conversely, parents’ height, genetic target height (for boys: Height (cm) = (mother's height + father's height + 13)/2, for girls: Height (cm) = (mother's height + father's height—13)/2), and sleep duration of 8–10 h (compared to >10 h) are negatively associated with short stature. Notably, gestational age and allergy history show no significant correlation with the incidence of short stature in this population.

**Table 6:** Influencing factors of stunted growth in children aged 6–12 in Zhuzhou city.

Influencing factors	B	Wald	*P*-value	Exp (B)/95% confidence interval
Mother's height	−0.123	254.599	<0.001	0.884 (0.871–0.898)
Father's height	−0.107	223.012	<0.001	0.899 (0.886–0.911)
Birth weight		28.888	<0.001	
Birth weight <2.5 kg	0.915	21.333	<0.001	2.496 (1.693–3.680)
Birth weight 2.5–4 kg	0.264	2.763	0.096	1.303 (0.954–1.779)
Gestational age		1.300	0.522	
Gestational age <37 weeks	−0.024	0.018	0.894	0.977 (0.689–1.384)
Gestational age 37–40 weeks	0.099	0.536	0.464	1.104 (0.847–1.440)
History of allergies	0.152	3.635	0.057	1.164 (0.996–1.361)
Daily duration of exercise		8.877	0.012	
Daily duration of exercise <0.5 h	0.297	7.327	0.007	1.346 (1.085–1.669)
Daily duration of exercise 0.5–1 h	0.134	1.576	0.209	1.143 (0.928–1.409)
Sleep duration		5.092	0.078	
Sleep duration <8 h	−0.188	1.196	0.274	0.828 (0.591–1.616)
Sleep duration 8–10 h	−0.278	4.830	0.028	0.757 (0.590–0.970)
Father's educational background		10.340	0.006	
Father's educational background: associate degree or below	0.785	4.380	0.036	2.192 (1.051–4.570)
Father's educational background: Bachelor's degree	0.452	1.463	0.226	1.571 (0.756–3.266)
Mother's educational background		0.186	0.911	
Mother's educational background: Associate degree or below	−0.154	0.150	0.699	0.857 (0.392–1.872)
Mother's educational background: Bachelor's degree	−0.170	0.182	0.669	0.844 (0.387–1.838)
Gender as male	0.523	45.222	<0.001	1.686 (1.448–1.964)
Underweight (body mass index <P5)	0.820	36.626	<0.001	2.269 (1.740–2.959)
Annual household income below 48,000 yuan	0.590	40.082	<0.001	1.804 (1.503–2.165)

Additionally, we analyzed the relationship between annual household income and the prevalence of short stature using the 15,010 parent questionnaires with valid income responses. The chi-square test results indicated that the proportion of children with short stature was higher among households with an annual income below 48,000 yuan compared to those without short stature, and the difference was statistically significant (Table 7). Binary regression analysis also revealed that an annual household income below 48,000 yuan is an independent risk factor for short stature (Table 6).

**Table 7:** Chi-square test of the relationship between annual household income and short statue.

Group	*n*	Annual household income			
		Below 48,000 yuan	48,000–120,000 yuan	120,000–240,000 yuan	above 240,000 yuan
Short statue	599	170^a^, 28.38%	295^b^, 49.25	108^b^, 18.03%	26^b^, 4.34%
Non-short statue	14,411	2,598^a^, 18.03%	7,558^b^, 52.45%	3,414^b^, 23.69%	841^b^, 5.84%

Note: Groups marked with “a” and “b” indicate pairwise comparisons. Groups marked “a” and “b” show statistically significant differences (*P* < 0.001), while “b” and “b” indicate no significant differences.

## 4 Discussion

### 4.1 Analysis of the current height status of children aged 6–12 in zhuzhou

Compared to national averages, the heights at the P50 percentile level for boys and girls aged 6 to 12 in Zhuzhou are consistently below the national level before puberty, indicating that the overall height of children in Zhuzhou lags behind the national average of 2005. However, after approximately age 11 for boys and age 9 for girls, the heights in Zhuzhou exceed the national average. This could be associated with earlier onset of sexual development in children influencing the growth patterns.

In the 2005 national growth curves from nine cities, girls typically surpass boys in height around 10.5 years of age with boys catching up and surpassing girls after 12.5 years of age, reflecting a typical pattern of earlier onset of puberty in girls than boys by approximately two years. Comparatively in Zhuzhou, the average height of girls exceeds that of boys after 8.75 years.

Similar findings have been observed by other regions by researchers such as Zhang (12) in Hanzhong, Shanxi, where girls’ heights gradually exceeds boys’ height around age 9, and by Li in Changsha, where girls aged 6–14 show higher heights between ages 9 and 11 (12, 13). These observations may be linked to the global trend of earlier onset of puberty and increasing incidence of central precocious puberty (14–17). The 2022 Chinese expert consensus on the diagnosis and treatment of central precocious puberty has revised the diagnostic age for girls to 7.5 years, emphasizing the importance of monitoring sexual development in children during physical surveys (18). Early detection and intervention of precocious puberty are anticipated to positively impact final adult height outcomes.

### 4.2 Analysis of the current status of short stature disease and influencing factors in Zhuzhou children

The 2021 meta-analysis covering 20 provinces, municipalities, and autonomous regions in China revealed a national overall incidence of short stature is 3.2%, with rural children exhibiting higher incidence compared to urban children (4.7% vs. 2.8%). Regional disparities were notable, with the Southwest region reporting the highest incidence (5.2%) followed by Central China (2.3%), East China (2.9%), and the Northeast (0.6%) (1). In Zhuzhou, the overall incidence of short stature stands at 4%, slightly higher than the national average with urban and suburban incidences at 3.7% and 5.8%, respectively. Logistic regression analysis identified several factors influencing short stature including lower parental heights, shorter genetic target height, shorter sleep duration and lesser weekly exercise consistent with broader patterns observed in child growth dynamics. The odds ratio EXP(B) from logistic analysis highlighted birth weight under 2.5 kg as the most significant risk factor for short stature. International studies corroborate that low birth weight, associated generally with maternal gestational health, nutritional status, lifestyle, and fetal nutrition, increases the likelihood of shorter stature, underscoring the importance of maternal health monitoring during gestation and vigilance in monitoring the growth of low birth weight children (19, 20). Malnutrition is also a risk factor for stunting in children aged 6–12 years, consistent with the findings of EI-Shafie et al. (21) Strengthening nutritional management for primary school students may thus contribute to height growth. Additionally, lower paternal education and low household annual income emerged as a risk factor for short stature, aligning with finding from Huang's study (22). Studies suggest that lower paternal education correlates with higher screen time and reduced physical activity among children (23, 24). In contrast, families where the father has received higher education often enjoy better economic conditions and nutritional status which may promote better physical growth in children (The father's level of education may have a more significant impact on the incidence of short stature in children compared to the mother's educational level due to traditional family values in China, where men often hold higher family status).

This study did not find significant associations between preterm birth, allergic diseases, and short stature among children aged 6–12 in Zhuzhou. While asthma has been linked to short stature in some studies, specific dermatitis showed no correlation (25, 26). However, the study acknowledges limitations such as non-classification of allergic diseases in the questionnaire and potential biases due to cluster sampling and low response rates to parental questionnaires. Further studies should address these limitations to refine understanding and interventions aimed at reducing short stature incidence effectively.

## 5 Conclusion

In conclusion, the average height of children aged 6–12 in the Zhuzhou area remains below the national average of 2005. In addition, the incidence of short stature is higher than national level compared to the meta-analysis in 2021. To address this, it is essential to ensure that children engage in more than half an hour of physical exercise daily and achieve over ten hours of sleep per night. Additionally, early monitoring and management of growth and development, as well as prioritizing the monitoring and management of nutritional status in primary school students, are crucial for children with low birth weight. Enhancing health education for families with less educated fathers and initiating early monitoring and intervention for children with short-statured parents are also vital strategies. These measures are likely to improve the height outcomes for children in Zhuzhou.

## Data Availability

The raw data supporting the conclusions of this article will be made available by the authors, without undue reservation.
